# Traditional sheep breeding practices under agroforestry system of Gedeo zone, Southern Ethiopia

**DOI:** 10.1371/journal.pone.0269263

**Published:** 2022-06-09

**Authors:** Biruh Tesfahun Tezera, Demeke Haile Engidashet

**Affiliations:** Animal Science Department, Dilla University, Dilla, Ethiopia; Colorado State University, UNITED STATES

## Abstract

The study was undertaken in Gedeo zone with the aim to identify traditional sheep breeding practices. Three potential districts (Yirgachefe, Bule and Gedeb) were purposely selected from Gedeo zone. A total of 180 smallholder sheep farmers were selected at random to complete a structured questionnaire that had been pretested. A pairwise ranking tool was used to prioritize ranked data during focus group discussion. Descriptive statistics were generated using SPSS version 26, while indices were computed for the ranked data. Mating happens because most farmers in Gedeo zone (80%) own breeding rams that are mixed and run with ewe flocks. Fifty-two percent of farmers were certain that their breeding rams mate with neighboring ewes. Nearly 70% of farmers prevented unwanted sheep breeding in their flocks by castrating or isolating undesirable rams from the ewe flock. Undesired or old breeding rams were replaced either from the same flock (58.9%) or purchased from local markets (41.1%). Breeding flock selection was common in Gedeo zone, with 97.2% of farmers selecting breeding rams and 93.3% of farmers selecting breeding ewes. Genotype (Index = 0.26) and body conformation (Index = 0.20) were the primary and secondary criteria used to select breeding ewes. Likewise, confirmation (Index = 0.25), genotype (Index = 0.24) and lamb growth rate (Index = 0.19) were the top three criteria when selecting breeding rams. Approximately three-quarters of interviewed farmers culled sheep due to poor body condition (31.8%), old age (28.6%), sickness (22.7%) and sterility (15.6%). Castration of rams was more common in Gedeb (58.3%) and Yirgachefe (55%) districts, and it was done for fattening, controlling unwanted breeding, and improving temperament. The mean selection and castration age of breeding rams were 13.18 and 30.72 months. In general, sheep breeding strategies for Gedeo zone should take into account section preferences and basic traditional sheep breeding practices.

## Background

Ethiopia has enormous potential in sheep that are raised under different production systems. The country reached 42.9 million head of sheep, of which 70% are females, and 30% are males [1]. Over 99.5% of the sheep population in Ethiopia are native, while 0.41% and 0.08% are hybrids and exotic breeds [1]. The indigenous sheep breeds in Ethiopia resulted from many generations’ human and natural selection for survival under harsh environmental conditions, diseases and adapted to low-input systems [2]. Across various production systems, sheep play a multipurpose role such as a source of income, food, manure, skin, fleece, wool, cultural and risk buffer during crop failure [3–5]. In tropical countries like Ethiopia, sheep breeding strategies for smallholder production have received little attention [6]; as a result, sustainable sheep improvement programs have been lacking [7]. Due to significant differences in production and reproduction parameters [8], sustainable sheep breeding programs needed to be designed to the specific goals of the production system and should consider the breeding culture [9]. Gedeo zone is located in the Southern Nation Nationalities and People Region state and is well known in the country for its unique and complex home garden agroforestry systems [10]. Gedeo agroforestry is an agrosilvopasture type in which trees, crops, and animals are all part of the system [10]. Apart from the perennial plants used for cash and food, animals such as sheep are the vital component of the Gedeo agroforestry practices [11]. In Gedeo zone, sheep production is entirely under smallholder situations. Knowledge of sheep breeding practices and techniques is necessary in such type of production system in order to develop long-term genetic improvement schemes [7]. Moreover, sheep breeding practices are a prerequisite in defining breeding objectives of specific genetic improvement programs at the smallholder level [12]. Thus, the current study addressed the traditional sheep breeding knowledge needed to design a sustainable sheep breeding scheme in the Gedeo zone.

## Materials and methods

### Ethics statement

The study used survey data from the participants. Farmers were briefed on the purpose of the study and the value of the information they provided prior to the start of the survey. Farmers were told about the confidentiality of their information and their freedom to decline or withdraw from research. Farmers were assured that there was no risk of participating in the study. Farmers were also explicitly told about the anonymous handling of individual replies and appropriate data management. Participants stated that they were willing to participate in the survey through verbal consent. Within the study area, most participants were illiterate and could not provide written consent. Besides, survey studies were exempted from ethics approval at the College of Agriculture and Natural Resources of Dilla University.

### Description of the study site

The research was carried out in the Gedeo zone, southern Ethiopia which is located geographically around 5050’ 19" to 6022’ 12"N latitude and 380 05’ 47" to 380 26’17"E longitude. Its elevation varies between 1450 and 3200 m. It is normal to get bimodal rainfall from March to May and August to October. The yearly rainfall fluctuates between 1200 and 1800mm. The temperature is between 18 and 25°c [13].

### Sampling procedure

A cross-sectional survey was conducted to assess sheep breeding practices of farmers in Gedeo zone. A multi-stage sampling procedure was employed to get representative samples. First, three research districts from the Gedeo zone, namely Bule, Gedeb, and Yirgachefe, were chosen purposely for their abundance of sheep and producer density. Secondly, a purposive sampling technique included nine kebeles (smallest administrative unit), making sheep production potential as a criterion. Thirdly, simple random sampling procedure with lottery method was applied to take sheep farmers from each kebele for data collection. A pre tested questionnaire was used to collect data from a total of 180 sheep farmers. Pairwise ranking tool was used during focus group discussion.

The sample size was determined by Adam [14] using the following formula.

$$n=\frac{N}{1+N\varepsilon^{2}\;}$$

Where,

n = the sample size

N = the population size

ε = adjusted margin of error [$\varepsilon =\left(\frac{\rho e}{z}\right)$]

ρ = the number of standard deviations that would include all possible values in the range = 4

e = the degree of accuracy expressed as a proportion = 0.04

z = z-value for the selected alpha level or confidence level at 96.84% = 2.15

The total number of sheep producing farmers in the three research districts is 27,943. Using the aforementioned Adam’s adjusted formula, the sample size determined was 180; hence, 60 sheep farmers from each district were chosen to participate in the current study.

### Data analysis

Using SPSS(Statistical Package for Social Science) version 26, descriptive statics were generated for the majority of sheep breeding practices including sheep possession, mating systems, culling techniques, sheep breeding monitoring, and mean for castration and selection age. The following formula was adopted from [15, 16] and applied to compute indices for breeding ewe and ram selection criteria for the first three rankings, utilizing the determined ranking proportion of smallholder farmers for each criterion from SPSS.

$$Index=\sum_{n=1}^{3}w_{c}x_{nc}/\sum_{c}\sum_{n=1}^{3}w_{c}x_{nc}$$

Where w_c_ is the rank weight associated with criteria c (w1 = 3, w2 = 2, w3 = 1), X_nc_ is the proportion of smallholder farmers who ranked the c^th^ criteria in the n^th^ rank (n = 1 to 3 ranks).

## Results and discussion

### Sheep possession

Table 1 shows the percentage of farmers who own sheep in each of the research districts. Almost all (99.44%) of respondents have kept adult ewes. Adult (breeding) rams, on the other hand, were owned by 80% of farmers. None of respondents have kept castrated rams in Bule district. Farmers kept almost similar number of male and female sheep between the ages of 6 and 12 months (65%).

**Table 1:** Percent possession of sheep by respondents.

Flock structure	Yirgachefe	Bule	Gedeb	Overall
	N(%)	N(%)	N(%)	N(%)
Adult ewes	60(100)	60(100)	59(98.33)	179(99.44)
Adult (breeding) rams	43(71.66)	55(91.66)	46(76.66)	144(80)
Castrated	22(36.66)	-(-)	11(18.33)	33(18.33)
Male (6–12 months)	39(65)	33(55)	45(76.67)	117(65)
Female (6–12 months)	38(63.33)	46(76.67)	34(56.67)	118(65.56)
Lambs(<6 months)	45(75)	51(85)	53(88.33)	149(82.78)

N: Number of respondents

### Sheep mating system

Farmers owing breeding rams in their flock further explained about the mating pattern. Most sheep farmers in Yirgachefe (86%) and Gedeb (58%) districts stated their breeding rams mate their own ewes as well as those of their neighbors (Table 2). More than one third of sheep farmers in Bule and Gedeb didn’t determine if their breeding rams only mate with ewes of their own flock or ewes of their neighbors’ flock.

**Table 2:** Farmers’ breeding ram mating patterns.

	Yirgachefe	Bule	Gedeb	Overall
	N(%)	N(%)	N(%)	N(%)
Only own flock	6(13.95)	18(32.7)	2(4.3)	26(18.49)
Own and neighbor flock	37(86.05)	18(32.7)	27(58.7)	82(56.16)
Undetermined	-(-)	19(34.6)	17(37)	36(25.34)

N: Number of respondents

Farmers usually mix breeding rams with ewe flock across all study districts (60% Yirgachefe and Bule, 68% Gedeb); as a result, mating happens (Table 3). In comparison to other study districts, farmers in Yirgachefe seldom released rams to the ewe flock for a fixed period of time. Farmers in Yirgachefe have been known to breed ewes only during a particular mating time.

**Table 3:** Farmers’ systems of sheep mating.

Sheep mating systems	Yirgachefe	Bule	Gedeb	Overall
	N(%)	N(%)	N(%)	N(%)
Always mix rams with ewe flock	36(60)	36(60)	41(68.3)	113(62.8)
Rams mate ewes during breeding time only	23(38.3)	13(21.7)	3(5)	39(21.7)
Rams released to ewe flock in determined period	1(1.7)	11(18.3)	16(26.7)	28(15.6)

N: Number of respondents

More than half of sheep producers in all study districts were sure that their breeding rams mate with neighboring ewes. A quarter of interviewed farmers were not certain to tell the mating scheme of breeding rams. Thus, breeding rams were not constantly used for a particular flock, i.e. any breeding ram had a chance to mate any ewe from any other flock in the village or at grazing. Similarly, Farmers in the western Amhara region use rams from grazing areas and borrow breeding rams from neighbors to mate ewes [7]. From the surveyed districts, it was well understood that most (62.8%) of farmers constantly mix breeding rams with ewes during herding and sheltering; therefore, mating occurs. Similar to the current finding, farmers in Horro district mix sheep in communal flocks; hence mating took place at random [17].

### Monitoring of sheep breeding

The majority of farmers in Yirgachefe (78.3%) and Gedeb (73.3%) and over half portion of farmers in Bule (56.7%) controlled undesired breeding in sheep (Table 4). Separation of undesirable rams from the ewe flock was a vital approach in Bule, whereas castration of undesired rams was a common technique in Yirgachefe to prevent unintended breeding. In Bule, very few farmers sell undesired rams to remove them from the breeding flock. Overall, nearly 70% of farmers were controlled undesired sheep breeding in Gedeo zone. This result contrasts with other parts of the country; northwest highlands [15], Setit Humera [18], North Wollo [19] and Metema district [20], where most of the farmers use uncontrolled breeding. Gedeo zone is recognized for its prominent agroforestry systems with high population density, a km^2^ of land hosting up to 1300 persons, the highest rural population density in Africa [11]. As a result, the community herding system was restricted, and sheep herders were obliged to rely heavily on sheep tethering. Thus, farmers got an opportunity to control their sheep mating system. Farmers protect undesired mating of both within and between flocks. In some production systems of Ethiopia, like the current, controlled mating is essential to match lambing time with a wet season and to avoid indiscriminate breeding [21]. Farmers in the current sheep producing area typically adopt one of two methods to prevent unwanted mating within the flock: castration or separation of undesired rams from the ewe flock.

**Table 4:** Methods to control undesired sheep breeding in Gedeo zone.

		Yirgachefe	Bule	Gedeb	Overall
		N(%)	N(%)	N(%)	N(%)
Farmers controlling undesired breeding		47(78.3)	34(56.7)	44(73.3)	125(69.4)
Farmers not controlling undesired breeding		13(21.7)	26(43.3)	16(26.7)	55(30.6)
Methods to control undesired breeding	Separating rams from ewe	18(38.3)	26(76.5)	24(54.5)	68(54.4)
	Castrating undesired rams	29(61.7)	6(17.6)	20(45.5)	55(44)
	Selling unwanted rams	-(-)	2(5.9)	-(-)	2(1.60)

N: Number of respondents

In contrast, about 30% of farmers didn’t control undesired sheep breeding (Table 4). The absence of breeding rams and the collective sheep flock raising system were revealed to be the two main culprits (Table 5). Similar reasons were reported for Wolayita zone [22] and Setit Humera [18]. Despite the fact that the collective herding system permits breeding females from different flocks to be mixed with breeding males from other flocks and minimizes inbreeding [6], it was only used by a few farmers in the study districts.

**Table 5:** Causes of uncontrolled breeding.

Causes	Yirgachefe	Bule	Gedeb	Overall
	N(%)	N(%)	N(%)	N(%)
Collective sheep raising system	10(76.9)	21(80.8)	14(87.5)	45(81.8)
Absence of breeding ram	3(23.1)	5(19.2)	1(6.3)	9(16.4)
Limited awareness	-	-	1(6.3)	1(1.8)

N: Number of respondents

### Replacements for breeding rams

Two key sources for breeding ram replacement were noted in the current study. Breeding rams descended from the same flock as ram lambs; about 58.9% of farmers reared ram lambs to replace breeding rams (Table 6). On the other hand, farmers purchased rams from local markets for breeding purposes. Similar reports were released in different parts of the country; Gamogofa zone [23], northwest highlands of Ethiopia [15], Setit Humera and Kafta Humera [24], Doyogena district [25] and Horro district [17].

**Table 6:** Sources to replace breeding ram.

	Yirgachefe	Bule	Gedeb	Overall
	N (%)	N (%)	N (%)	N (%)
Own flock	39(65)	35(58.3)	32(53.3)	106(58.9)
Market	21(35)	25(41.7)	28(46.7)	74(41.1)

N: Number of respondents

### Sheep selection practices

Most of the sampled farmers practiced selection of breeding stock as parents of the next generation; 97.2% select breeding rams, and 93.3% select breeding ewes (Table 7). The practice of ewe selection in the current study was comparable with Adiyo Kaka and Horo district, where 94.7% of the farmers selected breeding females [17]. However, selections of breeding rams have been better practiced than Menz and Afar [26], Habru and Gubalafto [27], Meket district [28] and northwest highlands of Ethiopia [15].

**Table 7:** Selection practices on ewes and rams.

	Yirgachefe	Bule	Gedeb	Overall
	N(%)	N(%)	N(%)	N(%)
Select ewes	58(96.7)	58(96.7)	59(98.3)	175(97.2)
Do not select ewes	2(3.3)	2(3.3)	1(1.7)	5(2.8)
Select rams	59(98.3)	53(88.3)	56(93.3)	168(93.3)
Do not select rams	1(1.7)	7(11.7)	4(6.7)	12(6.7)

N: Number of respondents

#### Breeding ewe selection criteria

Farmers were asked to rank different criteria applied to select ewes based on the tendency of practice and importance. Table 8 below illustrates the calculated indices of various selection criteria used by farmers to select ewes. The genotype of selected ewe was the uppermost criterion to select breeding ewes in Yirgachefe (Index = 0.29) and Gedeb (Index = 0.30) district whereas twinning ability (Index = 0.25) in Bule. In Yirgachefe and Bule districts, the second criterion for selecting the best ewe was body conformation, while lamb growth rate was in Gedeb district.

**Table 8:** Indices of various criteria used to select best ewes in sheep reproduction.

Traits	Yirgachefe	Bule	Gedeb	Overall
Body conformation	0.25	0.21	0.15	0.20
Color	0.04	0.06	0.00	0.03
Lamb growth rate	0.09	0.07	0.17	0.11
Mothering ability	0.11	0.05	0.12	0.09
Milk production	0.12	0.00	0.05	0.06
Earlier age at first lambing	0.04	0.14	0.05	0.08
Short lambing interval	0.02	0.02	0.11	0.05
Genotype	0.29	0.20	0.30	0.26
Twining ability	0.04	0.25	0.05	0.11

When pooling the different ewe selection criteria of the study districts, genotype (Index = 0.26) and body conformation (Index = 0.20) were the primary and secondary criteria used by farmers in Gedeo zone. Traditionally, farmers perceive the genotype as the best breeding ewe of local breed having better performance in growth and appearance. Body conformation, in general, is about the appropriate size and body condition of selected ewes. The current result is congruent with Tigray [29] western Amhara, [7] Ethiopia’s northwest highlands [15] Gamogofa Zone [23] Horo, and Adiyo Kaka districts [17] where body size was the main or second most important criterion for ewe selection. However, the result contradict with [7] who reported lambing interval had prime importance when selecting breeding ewes in Wolayita zone. Other selection criteria such as lamb growth rate, mothering and twinning ability were also moderately considered. The least priority was given to milk production, coat color and lambing interval during ewe selection in the current study site.

#### Breeding ram selection criteria

In the same way, indices were calculated for several ranked criteria used to choose the best breeding ram, as shown in Table 9 below. Farmers from Yirgachefe and Bule districts first examine body confirmation to choose breeding ram with indices of 0.27 and 0.33, respectively. Farmers in Gedeb district place genotype at the first position during the selection of breeding ram while it was a second most important criterion for farmers in Yirgachefe district. Fast-growing lambs were also considered as good breeding rams in the study districts; it was a second important criterion in Gedeb and Bule, while it was the third criterion in Yirgachefe.

**Table 9:** Indices of various criteria to select best rams in sheep reproduction.

Traits	Yirgachefe	Bule	Gedeb	Overall
Body confirmation	0.27	0.33	0.17	0.25
Presence of horn	0.10	0.01	0.00	0.04
Color	0.08	0.11	0.00	0.07
Lamb growth rate	0.18	0.22	0.18	0.19
Mating potency	0.09	0.05	0.18	0.11
Genotype	0.22	0.21	0.30	0.24
Adaptation	0.02	0.02	0.09	0.04
Age at first service	0.05	0.05	0.08	0.06

Collectively, in all study districts, farmers gave due attention for body confirmation (Index = 0.25), genotype (Index = 0.24) and lamb growth rate (Index = 0.19) when selecting breeding rams. Body size or body conformation was among the top priority criteria for breeding ram selection in west Gojam [30], Tahtay Maychew [16], central zone of Tigray [31], Aba’ala, [32], and North Shoa [33]. Likewise, in Tigray region, fast-growing ram lambs were preferred for breeding and ranked as a secondary criterion [29]. Fast-growing lambs finally attain better body conformation (body size) due to the existence of moderate to a high genetic and phenotypic correlation between the two traits [34]. Unlike the current finding, farmers in west Gojam, central zone of Tigray and Aba’ala supported breeding ram selection with pedigree information [30–32]. Farmers in the surveyed district further examined breeding rams for their mating potency, coat color and age at first service with the least priority.

### Sheep culling practice

The majority of farmers across the study districts imposed culling on their sheep flock (Table 10). Almost all respondents in Gedeb district have reported their experience in culling rams (93.3%) and ewes (96.7%).

**Table 10:** Farmers’ sheep culling practice.

Sheep culling practice		Yirgachefe		Bule		Gedeb		Overall	
		Ewes	Rams	Ewes	Rams	Ewes	Rams	Ewe	Ram
Practice culling	N	50	42	46	40	58	56	154	138
	%	83.30	70.00	76.70	66.70	96.70	93.30	85.60	76.70
No culling practice	N	10	18	14	20	2	4	26	42
	%	16.70	30.00	23.30	33.30	3.30	6.70	14.40	23.30

N: Number of respondents

In Yirgachefe district, the two major reasons to cull rams or ewes were health problem and poor body condition (Table 11). However, in Gedeb, farmers cull sheep primarily due to old age. Rams that had mating difficulties were also culled in Bule and Gedeb districts. Across all study districts, about three folds of interviewed farmers expressed their experience in culling sheep for various reasons. It has marked variation in culling experience with northwest highlands of Ethiopia [15] and Doyogena district [25], where 97% and 90% of farmers practiced culling. Poor body condition (31.8%), old age (28.6%), sickness (22.7%) and sterility (15.6%) were the top reasons to cull ewes reported in the recent study. Similarly, in Borana [35] and Hawassazuria district [36], the main reasons for culling breeding females were health issues, old age, and sterility. Likewise, in Habru district, farmers culled sheep due to old age and poor fertility [19]. In the present study area, poor body condition (29.7%), mating difficulty (24.6%), and health problems (23.2%) were the top culling criteria for breeding ram. Poor body condition was also the first reason for culling breeding males in Tahtay Maychew [16] and Doyogena district [25]. Similar to the current finding, health problem was among the reasons to cull male sheep in Borana [35]. Understanding farmers culling strategy is vital to determine breeding and stock replacement rates [37]. Selling and slaughtering of culled animals were the common culling strategy in the recent study. These two culling strategies were reported similarly for Setit Humera and Kafta Humera [24], Aba’ala [32] and Tahtay Maychew [16].

**Table 11:** Causes for sheep culling.

Causes		Yirgachefe		Bule		Gedeb		Overall	
		Ewes	Rams	Ewes	Rams	Ewes	Rams	Ewe	Ram
Health problem	N	20	18	9	6	6	8	35	32
	%	40.00	42.90	19.60	15.00	10.30	14.30	22.70	23.20
Aging	N	5	10	22	8	17	12	44	30
	%	10.00	23.80	47.80	20.00	29.30	21.40	28.60	21.70
Sterility	N	1	-	14	-	9	-	24	-
	%	2.00	-	30.40	-	15.50	-	15.60	-
Poor body condition	N	23	13	-	14	26	14	49	41
	%	46.00	31.00	-	35.00	44.80	25.00	31.80	29.70
Low milk yield	N	1	-	1	-	-	-	2	-
	%	2.00	-	2.20	-	-	-	1.30	-
Undesired color	N	-	1	-	-	-	-	-	1
	%	-	2.40	-	-	-	-	-	0.70
Mating difficulty	N	-	-	-	12	-	22	-	34
	%	-	-	-	30.00	-	39.30	-	24.60

N: Number of respondents

### Castration practice

Castration of rams has been better practiced in Gedeb (58.3%) and Yirgachefe (55%) compared to Bule district, where 98.3% of farmers didn’t castrate rams (Table 12). The unfamiliar castration could be due to high off-take rates of ram lambs; large numbers of ram lambs were marketed from Bule district than any other district in the zone. The scale of castration practice in the two districts (Gedeb and Yirgachefe) was comparable with Farta and Laygaynt districts where 52.8% of farmers practiced castration [38] and Horro district where 58% sheep owners do castration [37]. However, it was practiced in a lower degree than Menz (96.7%), Afar (97.2%), and Bonga (98.2%) [37]. The whole interviewed farmers in Gedeb district castrate their rams with modern method of castration, with burdizzo (Table 12). This service is provided by veterinary extension workers in the government structure at various sites. Comparably, 97% of interviewed farmers in Yirgachefe district use burdozzo to castrate the rams. This result was in contrast to the majority of research findings, which claimed that the traditional method of castration was common in various parts of the country [7, 30, 39–41]. Due to rare castration practice at Bule district, the purposes of castration are summarized only for Yirgachefe and Gedeb district (Table 12). Most interviewed farmers in Gedeb (60%) and Yirgachefe (93.9%) castrate rams for fattening. The same purpose of castration was indicated for most farmers in Hawassa Zuria district [36], Western Amhara [7] and Arbaminch Zuria district [40]. Furthermore, a small percentage of farmers castrate rams to control undesired breeding and improve temperament (to tame animals). Similar motivations towards castration was reported for Farta district [7], Selale area [39] and Doyogena district [25]. Thus, the purpose of castration for Ethiopian sheep producing communities is either to improve fattening or to avoid unnecessary mating or both [37].

**Table 12:** Sheep farmers’ castration practices.

Castration practice		Yirgachefe	Bule	Gedeb	Overall
		N(%)	N(%)	N(%)	N(%)
Experience on castration	Had experience	33(55)	1(1.7)	35(58.3)	69(38.3)
	Had no experience	27(45)	59(98.3)	25(41.7)	111(61.7)
Methods of castration	Traditional	1(3)	-	1(1.47)	1(3)
	Burdizzo	32(97)	35(100)	67(98.53)	32(97)
Purpose of castration	To control undesired breeding	1(3)	-	-	1(1.4)
	For fattening	31(93.9)	-	21(60)	53(76.8)
	To improve temperament	1(3)	-	2(5.7)	3(4.3)
	All	-	-	7(20)	7(10.1)
	To control breeding and fattening	-	-	2(5.70)	2(2.9)
	For fattening & to improve temperament	-	-	3(8.6)	3(4.3)

N: Number of respondents

### Age at selection and castration of rams

Farmers at Yirgachefe district selected breeding rams at the earliest age than any other district. Rams were selected at an average age of 10.22 months in Yirgachefe, whereas at 13.04 and 16.43 months at Bule and Gedeb districts (Table 13). In contrast to selection, castration of rams in Yirgachefe district was done at an older age (35.27 months) than Gedeb district (26.63 months). The overall selection age of breeding rams in the current study was 13.18 months. Thus, males were selected at older ages than Bonga (7.5 months), Afar (7.5 months) and Menz rams (9.9 months) [37]. Rams in Gedeo were castrated at older ages (30.72 months) than Horro (17.8 months) and Washera sheep (9.2 months) [17, 30]. There were two main reasons given for the late castration age: one was to enable the ram to mature before castration, and the other was to use the ram for breeding purposes before castration. Similar castration practices have been reported for Doyogena district; rams used for breeding purposes were castrated at the mean age of 28.68 months [25].

**Table 13:** Selection and castration age of rams.

Age of ram at castration and selection		Yirgachefe	Bule	Gedeb	Overall
		Mean±SE	Mean±SE	Mean±SE	Mean±SE
Age of ram	At selection	10.22±.50	13.04±.65	16.43±1.29	13.18±.54
	At castration	35.27± 2.86	-	26.63± 1.67	30.72± 1.68
Principles to determine castration age	Time of maturity	17(51.52)	-	20(57.14)	37(54.41)
	Use of the ram for breeding	16(48.48)	-	15(42.86)	31(45.59)

N: Number of respondents; SE: Standard Error

## Conclusion

Sheep mating occurs in the study districts when breeding rams and ewes are combined during herding and sheltering. It was also usual to use breeding rams from grazing or from a neighbor in the village. Farmers were able to prevent undesirable sheep mating by castrating and separating unwanted breeding rams. Old or undesired breeding rams were replaced from the same flock or purchased from local market. Breeding rams and ewes were chosen mostly on the basis of genotype and body conformation. Farmers often culled sheep that were in poor physical condition, were old, or were diseased. Burdizzo castration was performed at an average age of 30.72 months to fatten the animals, restrict reproduction, and improve temperament.
